# Common genetic variants in the *FETUB* locus, genetically predicted fetuin-B levels, and risk of insulin resistance in obese Chinese adults

**DOI:** 10.1097/MD.0000000000009234

**Published:** 2017-12-15

**Authors:** Zhibin Li, Changqin Liu, Xiulin Shi, Zheng Chen, Dongmei Wang, Long Li, Yichang Tu, Mingzhu Lin, Suhuan Liu, Shuyu Yang, Xuejun Li

**Affiliations:** a Xiamen Diabetes Institute; b Epidemiology Research Unit, the First Affiliated Hospital; c School of Public Health; d Department of Endocrinology and Diabetes, the First Affiliated Hospital, Xiamen University; e Department of Endocrinology and Diabetes, the Teaching Hospital of Fujian Medical University, Xiamen, China.

**Keywords:** fasting insulin, fetuin-B, insulin resistance, Mendelian randomization analysis, single-nucleotide polymorphism

## Abstract

Elevated serum fetuin-B is suggested to be associated with insulin resistance, but it is unknown if this association is causal. The aim of this study was to explore the potential causal relationship between fetuin-B and insulin resistance.

We used Mendelian randomization analysis by incorporating information of genetic variants in *FETUB* and serum fetuin-B concentrations with insulin resistance in 1148 obese Chinese adults.

Common genetic variants (*FETUB* rs4686434, rs6785067, and rs3733159) were significantly associated with serum fetuin-B concentrations but not with insulin resistance. Higher serum fetuin-B levels were significantly associated with increased homeostasis model assessment of insulin resistance (HOMA-IR) (0.17 [95%CI: 0.01 to 0.32, *P* = .037] 10^−6^ mol IU L^−2^ higher per SD). However, Mendelian randomization analysis using 3 single-nucleotide polymorphisms as instrumental variables did not support a significant association between genetically predicted fetuin-B levels and HOMA-IR (−0.09 [95%CI: −0.62 to 0.44, *P* = .738] 10^−6^ mol IU L^−2^ lower per SD). The regression coefficients for measured and genetically predicted fetuin-B concentrations on HOMA-IR were significantly different (*P* <.001).

This study suggests the association between fetuin-B and insulin resistance may not be causal. Future studies on the nongenetic determinants of serum fetuin-B concentration to assess if such unmeasured factors may confound the association between fetuin-B and insulin resistance as well as more pathway analysis for this association are warranted.

## 1 Introduction

Insulin resistance has been shown to play important roles in the pathogenesis of metabolic syndrome, type 2 diabetes, and cardiovascular disease.^[1–3]^ Although established evidence has documented that nonalcoholic fatty liver disease (NAFLD) is closely associated with insulin resistance,^[4]^ the factors linking NAFLD to insulin resistance are not fully understood. Fetuin-B, which is secreted from the liver, is the second member of the cystatin superfamily of cysteine protease inhibitors.^[5,6]^ Fetuin-B shares some similarity in functional analysis with fetuin-A, which has been found to cause insulin resistance by activating Toll-like receptors and inducing inflammatory signaling.^[6–8]^ Meex et al^[9]^ recently reported that fetuin-B was increased in humans with liver steatosis and patients with type 2 diabetes, impaired insulin action in myotubes and hepatocytes, and caused glucose intolerance in mice. Our previous data also suggest an independent association between serum fetuin-B and insulin resistance, but whether fetuin-B is causally related to insulin resistance is currently unknown.

Mendelian randomization (MR) analysis which employs genetic variants as instrumental variables under stringent assumptions to assess if the effect of a risk factor on the outcome, even in the presence of unmeasured confounding, is causal has been widely used in observational epidemiology studies.^[10–12]^ Unlike the conventional observation studies which are subject to lots of confounding bias and reverse causality, MR approach using the genetic alleles, which are located randomly during conception and inherited independent of confounding variables as the instrumental variables, has been widely accepted for causality inference.^[11]^ The *FETUB* gene encodes the protein fetuin-B, located in the human chromosome 3q27.3 with 8 exons. Based on the baseline examination of our designed cohort study of 1523 community-living healthy obese Chinese adults (unpublished data), we previously found that serum fetuin-B level was positively correlated with intrahepatic triglyceride content, and elevated serum fetuin-B was independently associated with increased risk of insulin resistance in obese Chinese. Furthermore, we have also shown that the minor allele G for *FETUB* rs4686434 was significantly associated with decreased intrahepatic triglyceride content and might confer lower susceptibility of NAFLD in Chinese adults. In the present study, by incorporating all information of common genetic variants in the *FETUB* locus and serum fetuin-B concentrations with insulin resistance, we aimed to explore the potential causal relationship between fetuin-B and insulin resistance in obese Chinese adults by suing MR analysis.

## 2 Materials and methods

### 2.1 Study subjects

**Figure 1:**
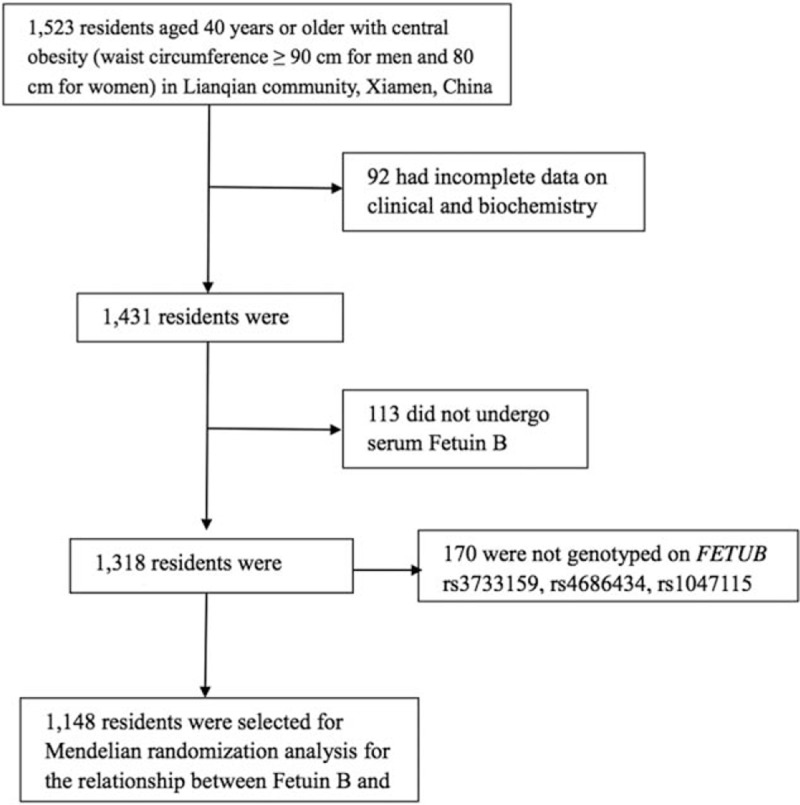
Study subjects selection diagram.

In 2011, a total of 1523 subjects aged ≥40 years living in Lianqian community, Xiamen, China with central obesity (waist circumference >90 cm for men and 80 cm for women) were recruited for the baseline examination of our designed cohort study. Details on subject sampling, recruitment, and evaluation have been described in our previous publications.^[13,14]^ Of them, 1148 (75.4%) subjects with the complete data on serum fetuin-B levels and genotypes on *FETUB* single-nucleotide polymorphism (SNPs) were kept for analysis (Fig. 1). This study was approved by the Human Research Ethics Committee of the First Affiliated Hospital of Xiamen University (Xiamen, China). Written informed consent was obtained from each participant.

### 2.2 Measurements

Standard questionnaires were used during face-to-face interview to collect sociodemographic status, lifestyle habits, present and previous history of health, and medications. Subjects were excluded if they had cancer, current treatment with systemic corticosteroids, biliary obstructive diseases, acute or chronic virus hepatitis, drug-induced liver diseases, total parenteral nutrition, autoimmune hepatitis, Wilson's disease, and known hyperthyroidism or hypothyroidism.

#### 2.2.1 Anthropometric measurements

Each subject underwent weight, height, and waist circumference measurements using a calibrated scale after removing shoes and heavy clothes. Body mass index (BMI) was calculated as weight in kilograms divided by height in squared meters. Body fat was quantified with the Hologic whole body DXA systems (Hologic Inc, Bedford, MA). Arterial blood pressure was measured with a mercury sphygmomanometer after sitting for at least 15 minutes. Three readings were taken at 5-min intervals and the mean of them was recorded.

#### 2.2.2 Biochemical measurements

Blood samples were obtained after 12-hour fasting and tested in the central laboratory of the First Affiliated Hospital, Xiamen University, Xiamen, China. Plasma glucose and serum lipid profiles, including triglyceride (TG), total cholesterol (TC), and high-density lipoprotein cholesterol (HDL-C) were determined on a HITACHI 7450 analyzer (HITACHI, Tokyo, Japan). Low-density lipoprotein cholesterol (LDL-C) was calculated by Friedewald's formula. Fasting plasma glucose (FPG) concentration was measured by the hexokinase method. Serum fasting insulin concentration was measured by an electrochemiluminiscence immunoassay (Roche Elecsys Insulin Test, Roche Diagnostics, Mannheim, Germany). Homeostasis model assessment of insulin resistance (HOMA-IR) was calculated using the formula: fasting serum insulin (mU/L) FPG (mmol/L) /22.5. And insulin resistance was defined as HOMA-IR ≥2.6×10^−6^ mol Meex IU/L^−2^.^[15]^

#### 2.2.3 Fetuin-B measurement

Serum fetuin-B concentration was measured using the enzyme-linked immunosorbent assay kits (Abcam, Cambridge, UK). The sensitivity of the assay was 4 ng/mL and the linear range of the standard was 4 to 50 ng/mL. The intra-assay variation was <10% and the interassay variation was <12%.

#### 2.2.4 Genotyping of the *FETUB* locus

Based on the publicly available phase III data of the International HapMap Project derived from the Chinese Han Beijing (CHB) population (release #28 August 2010), a genomic area on human chromosome 3q27.3 encompassing the complete *FETUB* gene (17.18 kb, 8 exons) as well as 5 and 3 kb of its 5′- and 3′-flanking regions, respectively, was screened. Within the *FETUB* locus, 7 HapMap SNPs were present and showed the Hardy–Weinberg equilibrium (HWE) (HapMap data), and 6 SNPs showed minor allele frequencies (MAFs) ≥0.05 and were genotyped in ≥50% of the HapMap individuals (HapMap CHB population, HapMap data). Based on Tagger analysis by using Haploview V.4.2 software (http://hapmap.ncbi.nlm.nih.gov/), 4 SNPs were selected as tagging SNPs covering all the other common SNPs within the locus with an *r*^2^ >0.8 (100% coverage): rs3733159 (T/G), rs4686434 (A/G), rs1047115 (A/C), and rs6785067 (G/A).

Genomic DNA was extracted from peripheral blood leukocytes using a commercial DNA isolation kit (*QIAamp DNA Blood Midi Kit*). The 4 *FETUB* tagging SNPs were genotyped by using the polymerase chain reaction method which was carried out using an annealing temperature of 65.0°C on the ABI3730XL DNA Analyzer.

### 2.3 Statistical analysis

Data was presented as mean ± standard deviation (SD) for continuous variable or number and percentage for categorical variable. Skewness and kurtosis test for normality of serum fetuin-B level was conducted and found it followed approximation of normal distribution. Differences between subjects (categorized by insulin resistance) were analyzed using one-way analysis of variance for continuous variable and χ^2^ test for categorical variable. HWE was tested using χ^2^ test (1 degree of freedom). Linkage disequilibrium (D′, *r*^2^) between the tested SNPs was analyzed using MIDAS v.1.0 (http://www.genes.org.uk/software/midas).^[16]^ Associations of the genotypes with serum fetuin-B concentration and insulin resistance were evaluated by coding the genotype with an additive model of inherence, that is, the genotype is coded with 0, 1, or 2 corresponding to the number of minor alleles carried by each subject. The mean fetuin-B concentration across genotypes and per minor allele was estimated in linear regression models with adjustment for age and sex.

Multivariable logistic regression was used to calculate adjusted odds ratio (OR) and 95% confidence intervals (CI) of genotypes and serum fetuin-B level for insulin resistance with adjustment for age, sex, educational level, ever smoking, ever drinking, regular physical exercise, waist, systolic blood pressure, diastolic blood pressure, TG, TC, and HDL-C. Genotypes were presented as subjects with 1, 2, or per 1 minor allele versus those with 0 minor allele. Based on screening 4 nonlinked tagging SNPs for *FETUB* in parallel, a *P*-value of .0125 was considered statistically significant according to Bonferroni correction for multiple comparisons.

To perform the MR analysis of fetuin-B and HOMA-IR, we first explored which of the 4 selected tagging SNPs within the *FETUB* locus were significantly associated with serum fetuin-B concentration, then 3 tagging *FETUB* SNPs (rs3733159, rs4686434, and rs6785067) which were found to be significantly associated with serum fetuin-B concentration were chosen as instrumental variables. Second, to test if assumptions required for MR analysis were met in our study, we explored if these 3 tagging SNPs were associated with any of the potential confounders listed in Table 1, and found no statistically significant association. Then we used the ordinary least square regression analysis for the association between measured serum fetuin-B concentration and HOMA-IR, and we found that per SD greater measured serum fetuin-B concentration was significantly associated with greater HOMA-IR. Furthermore, we used the 2-stage least squares approach (*ivregress* function in Stata) to estimate the difference in HOMA-IR per 1 SD difference in genetically predicted fetuin-B concentrations,^[17]^ in which an additive genetic model was used (i.e., fetuin-B concentration increased linearly with each additional minor allele of the genotypes). Lastly, the differences in HOMA-IR per 1 SD difference from analyses using measured serum fetuin-B concentrations and instrumentally predicted fetuin-B (i.e., endogenity) were compared using the Wooldridge test.

**Table 1:**
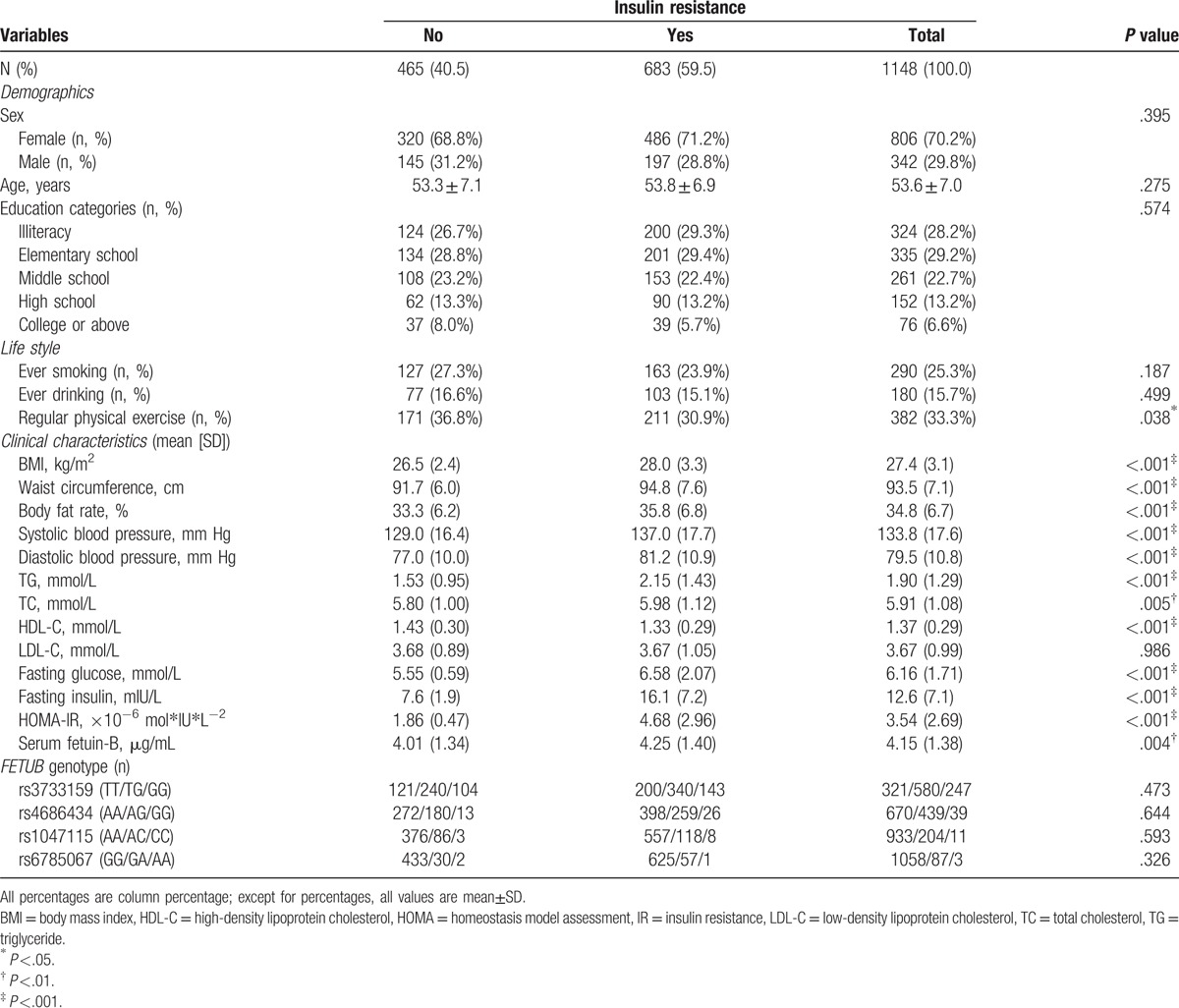
Demographic, lifestyle, clinical characteristics, and genotypes of subjects stratified by insulin resistance.

All *P*-values were 2-sided, and all statistical analyses were performed using Stata V.14.0 (StatCorp, College Station, TX).^[18]^

## 3 Results

### 3.1 Demographic and clinical characteristics stratified by insulin resistance

Of the 1148 subjects, 806 (70.2%) were females. And the prevalence rates of insulin resistance were 60.3% and 57.6% for females and males, respectively. Table 1 shows that, when compared with controls, subjects with insulin resistance have significantly higher levels of BMI, waist circumference, body fat rate, systolic and diastolic blood pressures, TG, TC, FPG, fasting insulin, and lower levels of HDL-C. Serum fetuin-B concentrations in subjects with insulin resistance were significantly higher than those without it (*P* = .004). There was no statistically significant difference in genotypes of 4 *FETUB* SNPs between subjects with and without insulin resistance.

### 3.2 Associations of *FETUB* SNPs with serum fetuin-B concentrations

Three of 4 *FETUB* tagging SNPs (rs3733159, rs1047115, and rs6785067) obeyed the HWE (*P* >.4), but *FETUB* rs4686434 significantly deviated from HWE (*P* = .001). Since no genotyping errors could be detected, we still included *FETUB* rs4686434 in our analyses. The MAFs of 4 tested SNPs ranged from 4.1% to 46.8% (Table 2). Results of the linkage disequilibrium (D′, *r*^2^) showed that the observed genetic linkage between the tested SNPs was low or moderate (*r*^2^ range: 0.05–0.45).

**Table 2:**
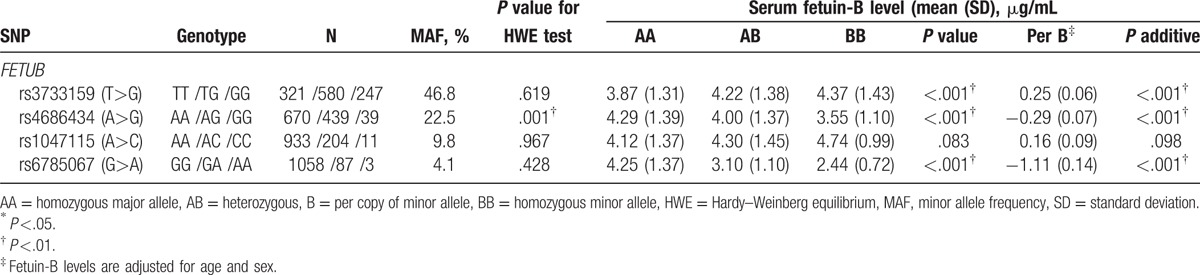
*FETUB* genotypes, MAFs, and serum fetuin-B levels according to *FETUB* genotype and per variant allele.

Serum fetuin-B concentrations according to *FETUB* genotypes are also shown in Table 2. Subjects carrying minor alleles of G in rs4686434 and A in rs6785067 showed significantly decreased serum fetuin-B concentrations. For minor alleles G in rs4686434 and A in rs6785067 after adjustment for age and sex, per minor alleles were associated with 0.29 (0.07) μg/mL and 1.11 (0.14) μg/mL (both *P*-values <.001) decreased serum fetuin-B, respectively. While subjects carrying minor alleles of G in rs3733159 showed significantly increased serum fetuin-B than their controls, with per minor allele G being associated with 0.25 ± 0.06 μg/mL increased serum fetuin-B (*P* <.001). Genotype of rs1047115 was not significantly associated with serum fetuin-B concentration.

### 3.3 Associations of *FETUB* SNPs and serum fetuin-B with insulin resistance

Adjusted ORs with associated 95% CI of *FETUB* SNPs and serum fetuin-B for insulin resistance are shown in Table 3. After adjusting for potential confounders (age, sex, educational level, ever smoking, ever drinking, regular physical exercise, waist, systolic blood pressure, diastolic blood pressure, TG, TC, and HDL-C), increased serum fetuin-B level was significantly associated with higher risk of insulin resistance, and the adjusted OR (95%CI) of per standard deviation (SD) increase of serum fetuin-B for insulin resistance was 1.16 (1.02 to 1.32, *P* = .020). Table 3 also showed that no significant association between *FETUB* SNPs and insulin resistance was found. Adjusted OR (95%CI) of per minor alleles of rs4686434, rs6785067, and rs3733159 for insulin resistance was 1.02 (0.81 to 1.29, *P* = .856), 1.19 (0.75 to 1.90, *P* = .461), and 0.89 (0.74 to 1. 07, *P* = .239), respectively.

**Table 3:**
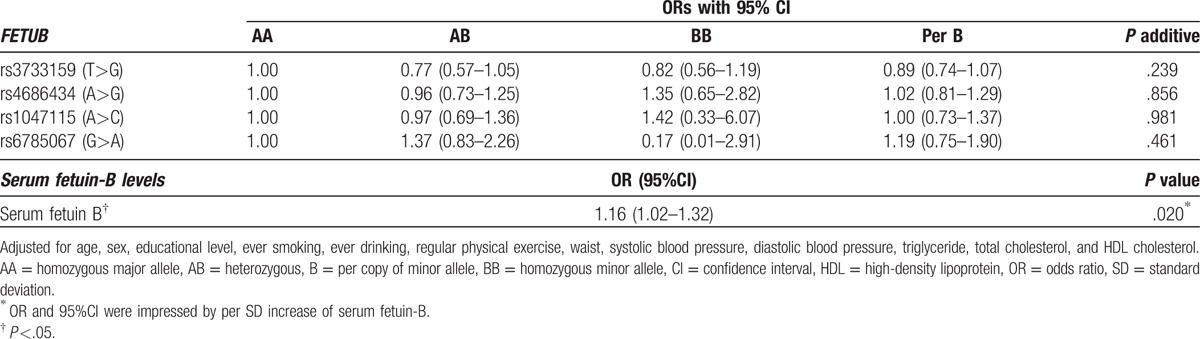
Associations of *FETUB* genotypes and minor allele, serum fetuin-B levels with insulin resistance.

### 3.4 *FETUB* SNPs, serum fetuin-B, and HOMA-IR

By using the ordinary least square regression analysis, per SD greater measured serum fetuin-B concentration was associated with 0.21 (95%CI: 0.05 to 2.07, *P* = .009) greater HOMA-IR (10^−6^ mol∗IU∗L^−2^) with adjustment for age and sex. After fully adjustment, per SD greater measured serum fetuin-B concentration was associated with 0.17 (95%CI: 0.01 to 0.32, *P* = .037) higher HOMA-IR (10^−6^ mol∗IU∗L^−2^).

To perform the MR analysis of genetically predicted fetuin-B with insulin resistance, 3 *FETUB* SNPs (rs3733159, rs4686434, and rs6785067) which were significantly associated with serum fetuin-B concentrations were chosen as instrumental variables and HOMA-IR was used as the outcome for maximal power. When using all the 3 *FETUB* SNPs (rs3733159, rs4686434, and rs6785067) as instrument variables simultaneously, per SD greater genetically predicted fetuin-B concentration was slightly associated with lower HOMA-IR (−0.09 [95%CI, −0.62–0.44] 10^−6^ mol IU L^−2^, *P* = .738), but it did not reach statistical significance. By using the Wooldridge test, we found that the regression coefficients for measured and genetically predicted fetuin-B concentrations on HOMA-IR were significantly different from one another (*P* <.001) (Table 4).

**Table 4:**
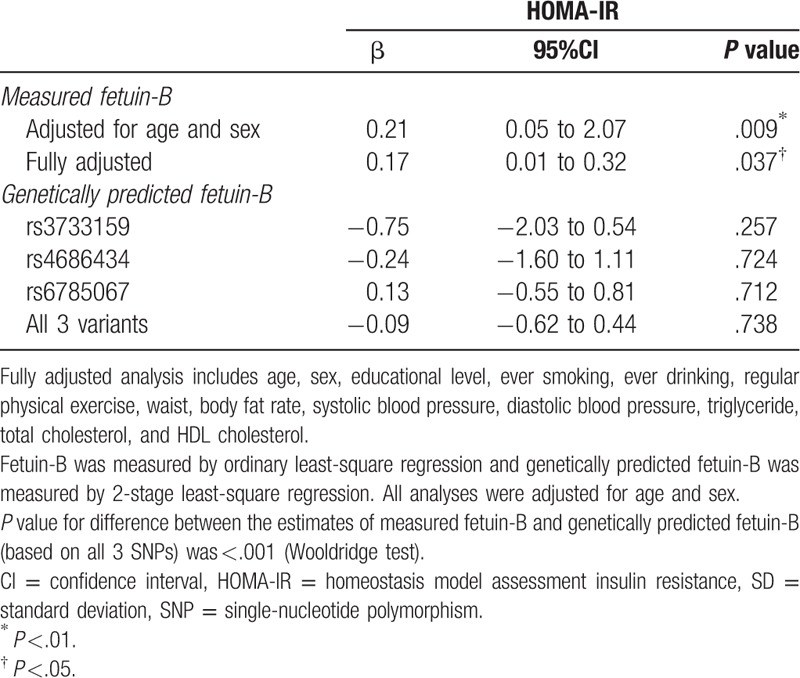
Differences of HOMA-IR per 1 SD difference in fetuin-B and per 1 SD difference in genetically predicted fetuin-B.

## 4 Discussion

The present study, to the best of our knowledge, is the first to examine the association between fetuin-B and insulin resistance using MR analysis. We firstly confirmed that increased serum fetuin-B level was significantly associated with higher risk of insulin resistance. Common genetic variants of *FETUB* SNPs (rs4686434, rs6785067, and rs3733159) were significantly associated with serum fetuin-B concentrations but were not associated with insulin resistance. By using the genetic variants of *FETUB* SNPs as instrumental variables to assess the potential causality of fetuin-B with insulin resistance, we found that the associations of measured fetuin-B and genetically predicted fetuin-B with HOMA-IR were significantly different. Therefore, the association between fetuin-B and insulin resistance may not be causal.

Denecke et al^[6]^ found that although fetuin-B shares some similarity with fetuin-A, the function of fetuin-B was not identical with that of fetuin-A. Fetuin-A has been found to cause insulin resistance, but the role of fetuin-B in the pathogenesis of insulin resistance has been seldom investigated after its identification. Meex et al^[9]^ reported that fetuin-B impaired insulin sensitivity in myotubes and hepatocytes, but unlike fetuin-A, it did not induce proinflammatory signaling. In mice, they found fetuin-B impaired significantly glucose tolerance but did not cause insulin resistance. However, when compared obese subjects with simple steatosis to those without steatosis, they found plasma fetuin-B correlated positively with fasting insulin and HOMA-IR.^[9]^ In the present study of obese Chinese adults, we consistently found that elevated serum fetuin-B level was independently associated with increased risk of insulin resistance after adjusting for potential confounding factors. Fetuin-A has been found to inhibit insulin receptor tyrosine kinase activity and promote inflammation,^[19,20]^ strengthen lipid-induced insulin resistance acting as an adaptor protein for saturated fatty acid-induced activation of Toll-like receptor 4 signaling^[7]^ and interact with free fatty acid to predict insulin resistance.^[8]^ However, the mechanisms underlying fetuin-B for insulin resistance have not been investigated well. Meex et al^[9]^ found fetuin-B had no effect on proinflammatory signaling or cytokine release, and they concluded that fetuin-B might induce insulin resistance in a manner quite distinct from that of fetuin-A.

To the best of our knowledge, there is no evidence available on genetic associations of variants among *FETUB* locus with serum fetuin-B concentrations and insulin resistance. We are probably the first to identify the minor allele G in rs4686434 and A in rs6785067 were significantly associated with decreased serum fetuin-B concentration, while the minor allele G for rs3733159 showed significantly increased serum fetuin-B concentration. We also found that there was no significant association between *FETUB* SNPs and insulin resistance in obese Chinese adults. Rather than being associated with decreased risk of insulin resistance as would be expected based on association observed between minor alleles of rs4686434 and rs6785067 with lower serum fetuin-B concentrations, these 2 minor alleles tended to be associated with increased risk of insulin resistance, although none reached statistical significance. How the *FETUB* SNP variants are likely to affect serum fetuin-B is currently unknown. It should be noted that our genetic association results are very preliminary and should be validated in different ethnic populations with much larger sample size.

In most observational studies, the direction of observed associations cannot be determined due to unmeasured confounding and the possibilities of reverse causation. One possible approach to strengthen causal inferences is to use instrumental variable if the randomized controlled trial is in unpractical situation. MR study is one form of instrumental variable analysis for observational studies which employs the germline genetic variants as instruments for environmental exposure and has been considered as analogous to randomized controlled trial.^[11]^ To perform MR analysis, a few stringent assumptions should be met well.^[21,22]^ First, the instrumental variable is associated with the risk of interest. Second, the instrumental variable is not associated with any confounding of the risk factor–outcome association. Third, the instrumental variable is conditionally independent of the outcome given the risk factor and confounding.^[23]^ In the present study, the *FETUB* SNP variants were found to be associated with serum fetuin-B concentrations only but not with any of the potential confounding factors or the outcome of insulin resistance. By using the *FETUB* SNP variants which were significantly associated with serum fetuin-B concentrations as instrumental variables to assess the potential causal association of fetuin-B with insulin resistance, we found that the associations of measured fetuin-B and genetically predicted fetuin-B with HOMA-IR were significantly different. Although we cannot exclude a potential association of genetically predicted fetuin-B with HOMA-IR, the estimate was significantly different from the positive association of the measured fetuin-B with HOMA-IR.

There are several possibilities for the discrepancy on the associations of measured fetuin-B and genetically predicted fetuin-B with HOMA-IR. First, our subjects are all adults with central obesity and older ages, and we may not have recruited appropriate population if the *FETUB* SNP variants affect insulin resistance earlier before they have been becoming old and obese. The second possibility of the discrepancy is that subjects who carry the risk alleles of *FETUB* SNP with elevated serum fetuin-B may have developed compensatory mechanisms against the risk of insulin resistance, as our obese subjects were all community-living and relatively healthy without apparently diagnosed diseases. The third explanation is that the observed association between serum fetuin-B concentrations and insulin resistance may be confounded by other determinants of fetuin-B and meanwhile *FETUB* SNP variants are not confounded by the same determinants. For example, fetuin-B is correlated with severity of hepatic steatosis which itself is also independently associated with insulin resistance. But the *FETUB* SNP variants have not been found to be associated with severity of hepatic steatosis, which has not been appropriately adjusted for in the multivariable analysis. Therefore, we could not exclude the possibility that the association of serum fetuin-B with insulin resistance is through other different mechanisms, and more pathway analysis for this association is warranted.

Although MR analysis that is less susceptible to confounding bias and reverse causality was used in the present study, a few limitations still need to be acknowledged. First, our subjects were all obese, and we may underestimate the association between serum fetuin-B and insulin resistance. Second, insulin resistance was determined by HOMA-IR calculated with fasting glucose and fasting insulin levels rather than the hyperinsulinemic euglycemic clamp, which remains the “gold standard” for accurately determining insulin resistance. Third, *FETUB* rs4686434 significantly deviated from HWE. But we believed this would not change our main findings, as the other 3 tagging SNPs (rs3733159, rs1047115, and rs6785067) obeyed the HWE, and there were no genotyping errors detected. Fourth, only 4 tagging SNPs were selected and rare SNPs with MAF <0.05 were not considered in the present study; the number of the genotyped SNPs was limited and explained only a small fraction (9.2%) of serum fetuin-B variation. Therefore, we cannot exclude the possibility that some rare SNPs among the *FETUB* locus are associated with insulin resistance and may underestimate the true association between genetically predicted fetuin-B levels and HOMA-IR. So for future studies on the possible causal relationship between fetuin-B and insulin resistance using MR analysis, more genetic variants, including both common and rare SNPs, should be included as the instrumental variables. And the fifth limitation was that we have not replicated our findings, as there was no another independent cohort available for us at present. Therefore, our results should be confirmed in different populations with much larger sample size in future.

In conclusion, the present study provided for the first time that the genetic variants in the *FETUB* locus were significantly associated with serum fetuin-B concentrations but were not associated with insulin resistance. Although we found that the elevated serum fetuin-B concentration was significantly associated with increased risk of insulin resistance, results from MR analysis suggested that this association may not be causal. Future studies on the nongenetic determinants of serum fetuin-B concentration to assess if such unmeasured factors in the present study may confound the association between fetuin-B and insulin resistance are needed.
